# ‘Part of the team as opposed to watching from the outside’: Critical incident study of autistic veterinary surgeons’ workdays

**DOI:** 10.1002/vetr.4957

**Published:** 2024-12-02

**Authors:** Kirstie Pickles, Jonathan Houdmont, Femke Smits, Bradley Hill

**Affiliations:** ^1^ School of Veterinary Medicine and Science University of Nottingham Sutton Bonington UK; ^2^ School of Medicine University of Nottingham Nottingham UK; ^3^ Harper Keele Veterinary School Keele University Newcastle‐under‐Lyme UK

**Keywords:** autism, clinical practice, critical incident study, neurodiversity awareness, veterinary surgeons

## Abstract

**Background:**

Autistic individuals experience differences in sensory processing, communication and executive function, which may affect their experience of the workplace. We investigated UK‐based autistic clinical veterinary surgeons’ experiences to establish contributing factors to a good or difficult workday.

**Methods:**

Purposive sampling was used to conduct semi‐structured qualitative interviews with 15 autistic veterinary surgeons. A critical incident technique was applied to explore the characteristics of a good and a difficult workday. Reflexive thematic analysis was used to identify recurrent themes and sub‐themes in the narratives.

**Results:**

Five major themes were identified as contributing to a difficult workday for autistic veterinarians: professional interactions, feeling out of control, the physical environment, role‐specific challenges and self‐doubt. Four overarching themes were identified as being associated with a good workday: positive interactions, feeling in control, having enough time and a sense of achievement.

**Limitations:**

As this is a qualitative study with a small number of participants, the extent to which these findings reflect the experience of the wider autistic veterinary surgeon community is unclear.

**Conclusion:**

Strategies to mitigate the effect of the most cited factors leading to difficult workdays, centring around neurodiversity awareness of colleagues and control of work, are likely to be helpful to autistic veterinary surgeons.

## INTRODUCTION

Autistic individuals are reported to be attracted to professions requiring technical skills.^1^ Similar to human medicine,^2^ autistic adults may be over‐represented in veterinary medicine due to autistic traits, such as monotropism (tendency to focus attention on a singular or small number of interests), attention to detail and systemising, being valued within the profession. However, these and other autistic traits, such as differences in sensory processing, communication styles and executive function, may elicit challenges in the workplace.^3^, ^4^, ^5^, ^6^

We have previously reported the mental wellbeing of autistic veterinary surgeons in the UK to be considerably lower than that of the general UK population of veterinary professionals and similar to that of the wider autistic adult population.^7^ Almost four‐fifths of autistic veterinarians reported probable or possible depression, with 44% of the variance in mental wellbeing being associated with key psychosocial workplace stressors delineated by the UK Health and Safety Executive. Similarly, autistic doctors report many challenges and stigma in the workplace, low disclosure rates and poor mental health.^6^ Indeed, a survey of 225 autistic doctors documented that three‐quarters of respondents had considered suicide (77%), one quarter had attempted suicide (24%) and half had engaged in self‐harm (49%).^6^ However, these workplace challenges are not limited to medical professions, with autistic performing artists also reporting difficulties and lack of awareness from colleagues and employers.^8^

In the UK, autism is covered by the Equality Act 2010, which requires employers to apply reasonable accommodations to remove or reduce a disadvantage related to an employee's disability. As autistic differences may result in workplace stress, identification of autism‐associated factors contributing to good and difficult workdays is a prerequisite of providing successful reasonable adjustments. The critical incident method^9^ can be used for this purpose and is defined as ‘a set of procedures for collecting direct observations of human behaviour in such a way to facilitate their potential usefulness in solving practical problems and developing broad psychological principles’.^10^ Given the current recruitment and retention crisis within the UK veterinary profession^11^ and demonstrated linkages between mental wellbeing and intention to leave the profession,^12^ further exploration of the experiences of autistic veterinary surgeons is warranted.

### Aim of the study

The purpose of this study was to use critical incident analysis to identify factors associated with good and difficult workdays for autistic veterinary surgeons in clinical practice in the UK.

## METHOD

### Participants and procedure

Fifteen interviews were conducted in the first quarter of 2022. The eligibility criteria comprised (1) a medical diagnosis of autism spectrum condition, (2) being at least 2 years postgraduation and (3) currently working in or having experience within the last 2 years in UK clinical practice. The exclusion criteria included any other co‐occurring neurodivergent condition (e.g., attention deficit hyperactivity disorder) and self‐identification. Participants were recruited via social media, including ‘neurodiverse vets’, ‘vets stay, go, diversify’, ‘veterinary voices’, ‘veterinary spoonholders’ and British Veterinary Ethnicity and Diversity Society Facebook pages. Recruitment letters were also published in the *Veterinary Times* and *Veterinary Record*.

The number of interviews conducted was dictated by the point at which thematic saturation was achieved and the timeframe available for the study. The participants could choose not to answer any question(s) or withdraw from the study at any point and no incentives were offered.

### Critical incident interview

The interview (Appendix A, Supporting Information) focused on exploring both a good and a difficult day at work. Interview questions had been pilot tested on the authors’ colleagues, including autistic individuals. To facilitate recall, participants were emailed 2 days prior to the interview to prompt them to reflect on the characteristics of a good and a difficult workday.

Initial ‘ice‐breaker’ questions established participants’ demographics, including the type of clinical work they were engaged in, year of graduation, when they received an autism diagnosis and what led them to seek a diagnosis. The participants were then asked to describe a specific good workday and the factors that contributed to it. They were also asked to describe a difficult workday and the events that caused them to perceive it in this way. At the end of the interview, the participants were given the opportunity to share any further pertinent information.

The interviews were conducted via video call in Microsoft Teams and automatically generated transcripts were manually checked against the video recordings for accuracy. Prior to analysis, any identifying material was removed, and each transcript was allocated an identification number.

### Analytical approach

Data were analysed in NVivo 12 (QSR International, 2020) using reflexive thematic analysis,^13^ which is commonly used in workplace health and wellbeing research to identify common themes and patterns in qualitative datasets.^14^, ^15^ The notion of reflexivity is important here because it positions the researcher as active in the analytical process. Moreover, it offers a pragmatic solution to research questions that is capable of addressing what Braun and Clarke^13^ refer to as ‘positions of fluidity and messiness … [that] … are often not binaries, but rather continua, and/or have scope for blurring’.

Two thematic analyses were conducted, one for characteristics of a good workday and another for characteristics of a difficult day. Following immersion in the data, coding of words or phrases that reflected work characteristics experienced by autistic veterinarians was conducted using both inductive and deductive analysis. From these codes, candidate themes were developed, that is, broad organising concepts that unified codes that recurred in the data and had meaning in relation to the research questions. Finally, candidate themes and sub‐themes were reviewed across transcripts to ensure that they accurately reflected the data before being defined and named. Primary coding of themes was performed by FS and then reviewed by the remaining authors to assess its adequacy and accuracy, with KP and BH providing veterinary and autistic context where required. All authors reviewed the themes and their descriptors. The thematic structure was refined and finalised through discussion, with all authors agreeing with the final themes. Content analysis was also performed by counting the frequency of themes in order to identify their relative prominence in the data. These frequencies are presented only to support the strength of the findings in these data rather than to estimate prevalence more generally. The speech filler has been removed from all direct quotes and grammatical edits are minimal, only made when necessary for comprehension. As themes were common across all participants and to ensure anonymity, participant identifying information was removed and names were replaced with a participant identification number.

The interviewer was a female veterinary student (FS) conducting the study in fulfilment of a Master's degree by research. The participants may have felt more comfortable discussing their experiences with an individual outside of the mainstream veterinary profession, which is often perceived as small and interconnected. However, being non‐autistic and a student, the interviewer could also not fully relate to their experiences, which may have affected interpretation of the interviews. Two authors (KP and BH) are autistic veterinary surgeons and their lived experience may have influenced interpretation of the data. Discussion of data and themes as a team was used to ensure that all viewpoints were considered.

## RESULTS

### Participant characteristics

A total of 15 interviews lasting 20‒49 minutes were conducted. The social and occupational demographic characteristics of interview participants are shown in Table 1.

**TABLE 1:** Social and occupational demographic characteristics of the interview participants.

	*N* (%)
Gender	
Male	3 (20.0%)
Female	12 (80.0%)
Job category	
Companion animal	8 (53.3%)
Farm animal	2 (13.3%)
Equine	2 (13.3%)
Mixed	1 (6.67%)
Other	2 (13.3%)
Years in practice	
≤9	3 (20.0%)
10‒19	3 (20.0%)
20‒29	5 (33.3%)
≥30	3 (20.0%)
Not specified	1 (6.67%)
Years since diagnosis	
<1	5 (33.3%)
1‒5	8 (53.3%)
6‒10	0 (0.00%)
>10	2 (13.3%)
Not specified	0 (0.00%)

### Difficult workdays

**FIGURE 1:**
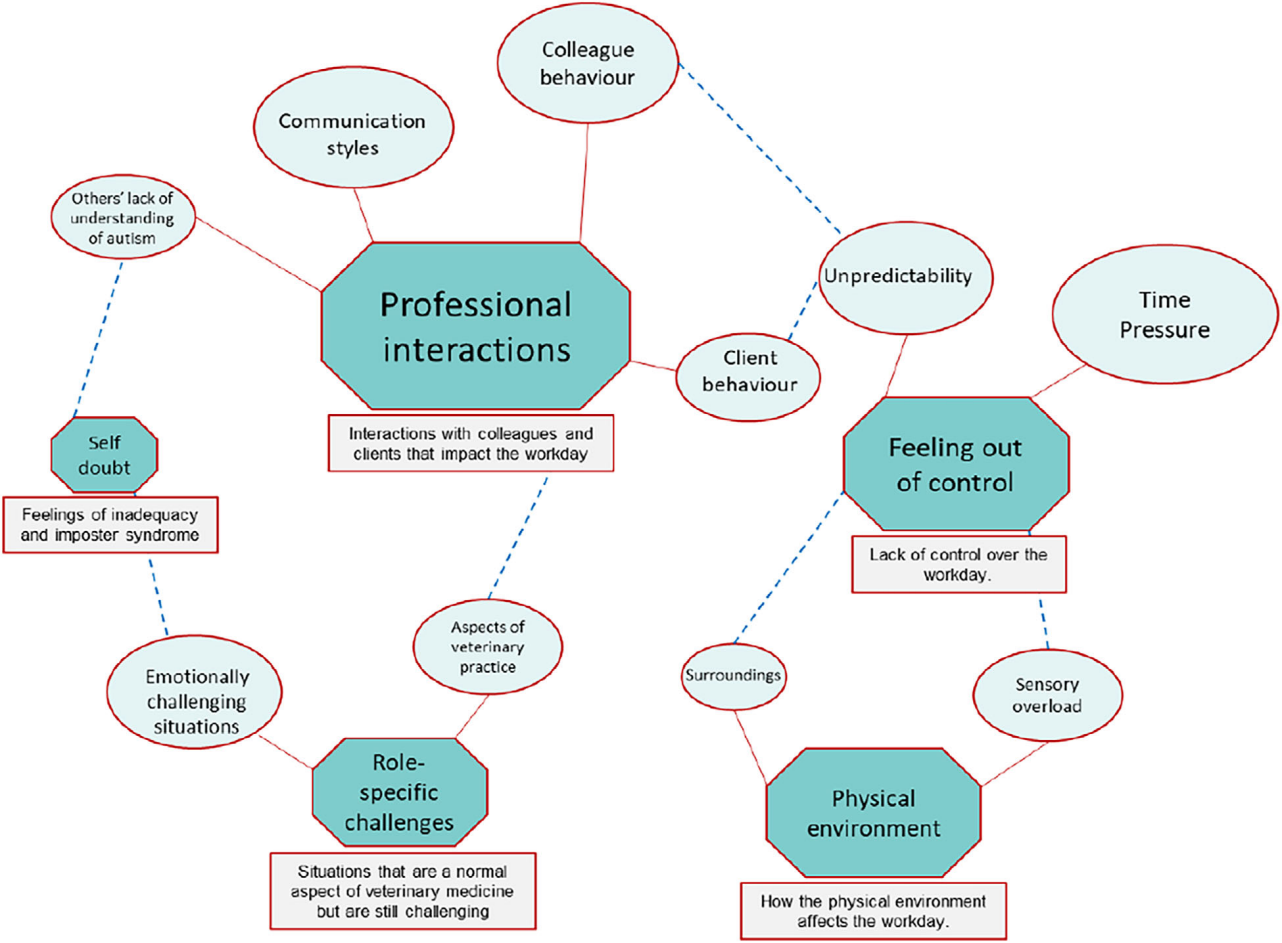
Thematic map representing a difficult day at work for autistic veterinary surgeons (*n* = 15). Key themes are shown in octagonal shapes with sub‐themes in oval shapes. The size of the shapes is proportionate to the number of times the theme was mentioned by the participants

Analysis of the individual coding statements (*N* = 323) identified five major themes and 10 sub‐themes contributing to a difficult workday for autistic veterinarians (Table 2 and Figure 1).

**TABLE 2:** Themes and sub‐themes associated with a difficult workday, including the number of respondents who mentioned each theme one or more times and the total number of referenced statements.

Themes and sub‐themes	Description	Example quote	No. of respondents mentioning this theme	Total statements mentioning this theme
Theme 1: Professional interactions	Interactions with colleagues and clients that impact the workday.	I've seen myself leaving the career just from some days of non‐stop talking and interactions.	15 (100%)	112 (37.7%)
Sub‐theme 1: Client behaviour	Incidences where the actions of the clients cause feelings of stress.	I guess [clients] being difficult or challenging me, or confrontational, that makes it more difficult and more stressful for me.	9 (60.0%)	20 (6.73%)
Sub‐theme 2: Colleague behaviour	Incidences wherein the behaviour of colleagues impact the workday.	It's a communication with colleagues thing, and anxiety about communication with colleagues, whereas I don't have that at all with clients.	14 (93.3%)	40 (13.5%)
Sub‐theme 3: Communication styles	Differences in communication styles between participants and their clients and colleagues and difficulties with these differences.	My nightmare would be our lunch break with everybody in the same room chatting away. I mean, I would be very stressed if that were to happen.	14 (93.3%)	51 (17.2%)
Sub‐theme 4: Others’ lack of awareness of autism	Both colleagues’ and clients’ lack of understanding and awareness of autism leads to a sense of ‘otherness’.	Most people seem to think that my being autistic was something I should hide, and I found that really frustrating because I can't be supported unless people know.	9 (60.0%)	19 (6.40%)
Theme 2: Feeling out of control	Lack of control over the workday.	If I'm talking about bad days, it's when it's full‐on constant stress, constant stuff, the whole day just feels like you're not done and it's just a little overwhelming.	14 (93.3%)	65 (21.9%)
Sub‐theme 1: Time pressure	Feeling rushed or pressed for time and changes to the schedule.	I get frustrated when things are expected of me in time that I don't have to give because that will stress me out.	14 (93.3%)	56 (18.9%)
Sub‐theme 2: Unpredictability	Unpredictable situations that cause unease.	You think you can do something and then suddenly someone rings up and changes the day completely on its head and it might be something that is unpredictable.	11 (73.3%)	31 (10.4%)
Theme 3: The physical environment	How the physical environment affects the workday.	Trying to find somewhere that's decent to sit that's not cold or wet or has wasps or has a phone or that has people running past all the time just makes things worse.	13 (86.7%)	37 (12.5%)
Sub‐theme 1: Sensory overload	Overwhelming sensory input in a practice environment.	But background noise, dogs barking and lack of quiet sometimes I think is just one of those things that kind of eats away underneath.	12 (80.0%)	35 (11.8%)
Sub‐theme 2: Surroundings	Organisation and layout of the surroundings.	I find it really sort of discombobulating when things aren't quite in the right place.	10 (66.6%)	15 (5.56%)
Theme 4: Role‐specific challenges	Situations that are a normal aspect of veterinary medicine but are still challenging.	A difficult day for me at work would be those high stakes, high pressured moments that are inevitable.	8 (53.3%)	45 (15.2%)
Sub‐theme 1: Emotionally challenging situations	Emotionally charged clinical cases.	You feel like, you know, you're just Doctor Death.	7 (46.7%)	26 (8.75%)
Sub‐theme 2: Aspects of veterinary practice	Typical aspects of practising veterinary medicine which increase stress levels.	I find on‐call quite difficult because it's obviously unplanned and in the middle of the night.	4 (26.7%)	20 (6.73%)
Theme 5: Self‐doubt	Feelings of inadequacy and imposter syndrome.	Going home from work, and thinking the job is not compatible with me as a human being and that somehow, I am a massive failure.	6 (40.0%)	16 (5.39%)

#### Theme 1: Professional interactions

This theme was defined as any communication or interaction during the workday and the interviewees’ perceptions of these interactions and comprised four sub‐themes: client behaviour, colleague behaviour, communication styles and others’ lack of autism awareness. Many interviewees described these interaction difficulties as increasing their stress levels, which made it more difficult to cope with other stressors.

Colleague behaviour was mentioned by all participants except one and described the negative impact of colleagues’ actions on the participant's day. Relationships and interactions with colleagues—both interactions that colleagues have with each other and colleagues’ behaviour towards participants—were reported to have a greater impact than interactions with clients: ‘It's a communication with colleagues thing, and anxiety about communication with colleagues, whereas I don't have that at all with clients’ (participant 1). Discriminatory behaviour was also described: ‘Most people seem to think that me being autistic was something I should hide, and I found that really frustrating because I can't be supported unless people know’ (participant 2).

Differences in communication and not understanding colleagues’ behaviour were frequently described: ‘My take on most of the people, neurotypicals if you like, is that they spend a lot of time trying to score points off each other and I just can't get my head around that’ (participant 12). Unnecessary interruptions from colleagues were also frequently reported: ‘I'm quite bad with getting interrupted all the time […] if it's just some random stuff that could have waited till the end of consults, I've lost my train of thought’ (participant 5).

Client behaviour was mentioned by 60% of participants. This included unpleasant behaviour and client complaints stemming from differences in communication, intersecting with the sub‐theme of others’ lack of autism awareness: ‘Instead of chatting, I just get the dog and vaccinate it … and get on to the next one. And there are some people who really, really don't like that, and have complained and made proper complaints’ (participant 6).

Communication styles were mentioned by all but one participant with differences in communication styles exacerbating stressful situations: ‘I find if I don't put on my vet head or put on my camouflage, I can inevitably end up saying something that will offend someone’ (participant 1). Phone consultations were stressful for some individuals: ‘I really, really hate speaking on the phone. … I had to make an official call yesterday and I was like shaking for half an hour’ (participant 7).

Differences in communication styles dovetail with the minor themes of colleague behaviour and others’ misunderstanding of autism. Missing nuances of colleagues’ communication and mutual misunderstanding were frequently described: ‘It goes right over my head, you will then feedback to somebody else that I didn't listen to you when I did listen to you, but what I heard was not what you said’ (participant 2). Direct communication and not valuing small talk is common to autistic people and was often reported to be misunderstood: ‘I can't do the chit chat stuff. I can talk work, but I can't do the gossipy stuff’ (participant 10). Quiet time away from people was used by some participants as a coping strategy: ‘I feel like I can be who I want to be …without having to put on a front’ (participant 1).

Others’ misunderstanding of autism was mentioned by 60% of participants, with managers and colleagues not understanding, or being ignorant of, their autistic traits and specific needs: ‘The worst days are when I open up to my colleagues about how hard a particular thing was, and then they brush it off’ (participant 1).

#### Theme 2: Feeling out of control

This overarching theme consists of two sub‐themes: time pressure and unpredictability. There are many unpredictable elements intrinsic to life in veterinary practice; however, this lack of control was reported to increase the stress levels of autistic veterinary surgeons.

Time pressure was defined as feeling rushed during the day and was identified by all but one participant as a substantial source of stress: ‘other people don't seem to realise how long each thing seems to take and they're pressurising you to do three things at once’ (participant 8). Over half (*N* = 9) mentioned that their most difficult days often did not include a lunch break, or at least not a restful one: ‘I would hate to walk into a staff room that was hustling and bustling and busy. I think that's just like a nightmare. It feels far too chaotic, feels like sensory overload’ (participant 1).

This sub‐theme also intersects with the theme of client behaviour, as there were reports of clients turning up late for appointments that impacted the schedule for the rest of the day: ‘The schedule went all wonky because somebody else turned up late, but they didn't turn up late enough to justify denying them a consult, so everything went south’ (participant 2).

Unpredictability was identified through reports of uncertain situations causing anxiety. In general, lack of control of situations made workdays more challenging: ‘I think the most important thing is having that control over what happens. When things go wrong, I don't have control over what happens’ (participant 15).

#### Theme 3: The physical environment

The physical environment consisted of two sub‐themes, sensory overload and surroundings, and intersected with theme 2, feeling out of control, as chaos and disorganisation in the participants’ environments increased feelings of loss of control.

Sensory overload includes factors related to sensory overstimulation and was reported by 80% of interviewees. Noise and strong smells were most frequently mentioned, with repetitive noises, such as beeping machines or phones ringing, being the most pertinent. For some, this was very overwhelming: ‘He barked very loudly and very high pitched, right at my left ear in a small echoey room, and honestly my vision went white for a second because it hurt so badly’ (participant 2).

Sensory overload also intersects with professional interactions as some people found ‘chatty’ colleagues or clients difficult or that a lot of ‘chaos’ significantly increased their stress levels: ‘I find interruptions hard or the kind of background noise and you know people just talking all the time like, you know, I like to work in quite quiet environments and focus’ (participant 3).

Surroundings involved comments regarding the cleanliness and organisation of the workplace. Needing a familiar, organised space to work in was very important to some participants: ‘Recently the nurses re‐organised one of the surgery rooms and put everything in bins, and I can't find anything now and they keep saying, oh no, it'll be fine you'll get used to it. I'm going, it's been three months now, I still can't find anything because you hid it all. Then, you didn't label the drawers’ (participant 2). Additionally, the layout and organisation of the practice itself increased feelings of stress for some interviewees. This intersected with professional interactions because poor environmental organisation or lack of space led to participants being unable to find a quiet place to work or have a restorative break: ‘We've got quite a small staff room and if there's more than three people in there, I don't stay, so I'll go back up and I work through [my lunch break] in front of my computer’ (participant 10).

#### Theme 4: Role‐specific challenges

This theme comprised two sub‐themes: emotionally challenging situations and aspects of veterinary practice. There are many unavoidable aspects of veterinary practice that can play a role in job stress. Role‐specific challenges intersected with professional interactions and feeling out of control as these contain job elements that are unavoidable; however, this theme encompasses the elements that make up the core of veterinary practice, primarily working with patients.

Emotionally challenging situations were difficult cases that did not go to plan or ended with the animal dying. Participants felt emotionally exhausted after a tough case due to having to compartmentalise their emotions on top of camouflaging their autistic traits: ‘Those high stakes, high pressured moments that are inevitable that people think I seem very calm and very relaxed and very professional, but in reality, I find them very, very difficult’ (participant 1).

Aspects of veterinary practice included out‐of‐hours commitments, difficulties with equipment and difficult cases: ‘We couldn't get (the x‐ray machine) to work and so then I get really, really stressed because this is a sick foal which is getting sicker by the minute, we're getting massively delayed ‘cause equipment doesn't work’ (participant 8).

#### Theme 5: Self‐doubt

Self‐doubt was mentioned by approximately half of the participants (*N =* 7). Several interviewees described feelings of ‘imposter syndrome’ whereby high‐achieving individuals fail to accept their accomplishments, despite their success, and experience consistent feelings of self‐doubt.^16^ Ruminating on colleagues’ comments or thinking that they were in the wrong was also described: ‘Every interaction with every individual is analysed and processed to determine whether I may or may not have done something to annoy or upset them’ (participant 13).

### Good workdays

**FIGURE 2:**
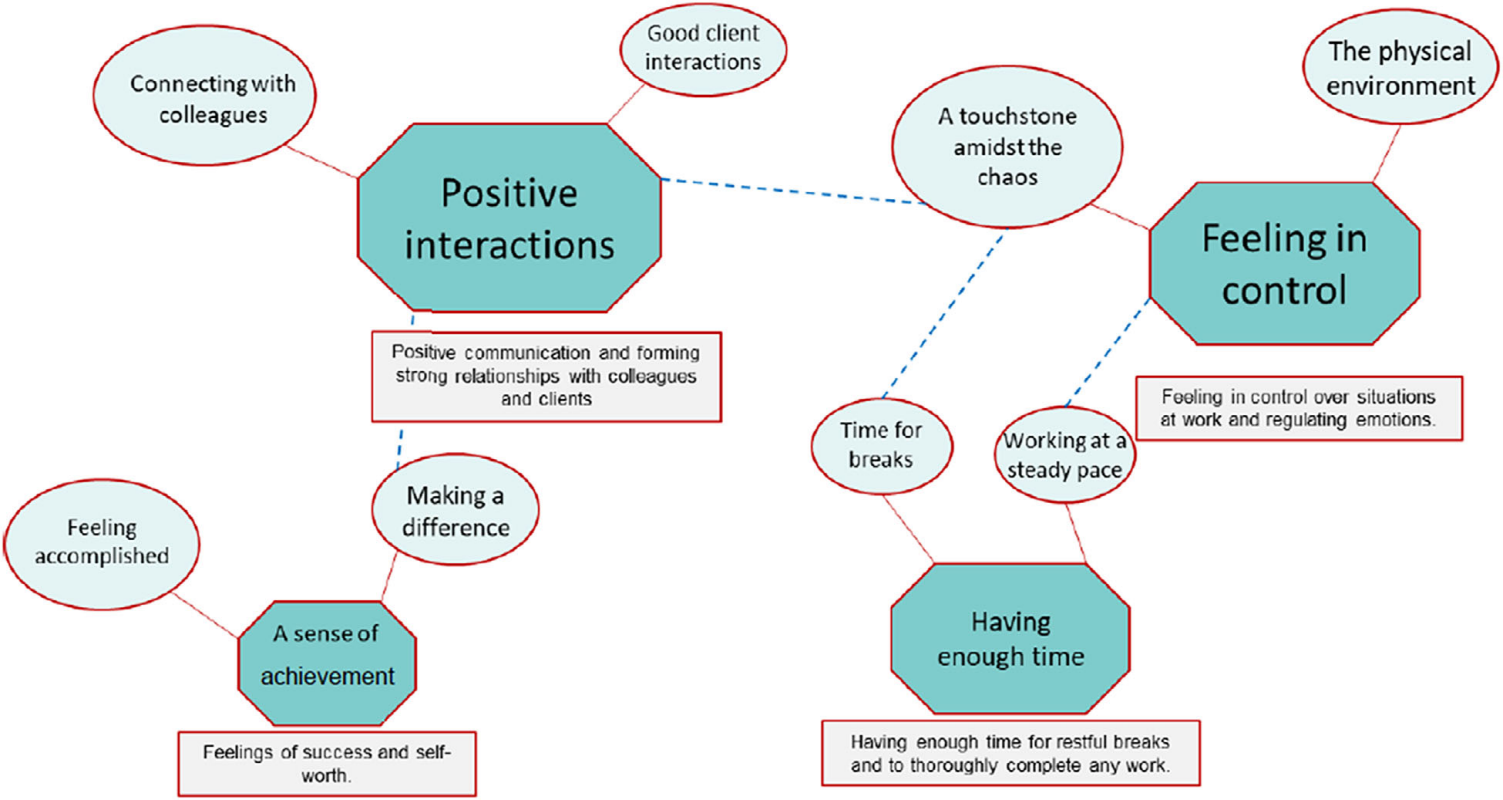
Thematic map representing a good day at work for autistic veterinary surgeons (*n* = 15). Key themes are shown in octagonal shapes with sub‐themes in oval shapes. The size of the shapes is proportionate to the number of times the theme was mentioned by the participants

Analysis of a good workday led to the identification of four overarching themes and eight sub‐themes from 169 individual references (Table 3 and Figure 2).

**TABLE 3:** Themes and sub‐themes associated with a good workday, including the number of respondents who mentioned each theme one or more times and the total number of referenced statements

Themes and sub‐themes	Description	Example quote	No. of respondents mentioning this theme	Total statements mentioning this theme
Theme 1: Positive Interactions	Positive communication and forming strong relationships with colleagues and clients.	Social interactions have always been a real problem for me but as a veterinarian its professional, it's much easier than social interaction.	15 (100%)	51 (32.5%)
Sub‐theme 1: Connecting with colleagues	Feeling part of the team and being able to connect with colleagues without feelings of social pressure.	It's nice when I get a chance to talk to my team during the day and I feel like I'm part of the team as opposed to watching from the outside.	14 (93.3%)	36 (22.9%)
Sub‐theme 2: Good client interactions	Easily and successfully communicating with clients during consultations.	The clients were all quite amenable. Everything was done in a good‐natured manner, there's no nastiness, no criticism, no recrimination, or anything.	11 (73.3%)	16 (10.2%)
Theme 2: Feeling in control	Feeling in control over situations at work and emotional regulation.	A day where everything goes to plan, where what walks through the door is roughly what you're expecting.	14 (93.3%)	63 (40.1%)
Sub‐theme 1: The physical environment	Having control over the working conditions and organisation of the physical environment.	I think controlling my environment makes a big difference. When I come in, I'll just tidy things up, then I don't have to think about them.	8 (53.3%)	17 (10.8%)
Sub‐theme 2: A touchstone amidst the chaos	Predictable and structured elements of the workday acted as a touchstone to help cope with difficult situations.	Vet medicine is never going to be predictable but keeping some things steady as a touchstone helps me at least deal with the chaos of the rest of the day.	14 (93.3%)	48 (30.6%)
Theme 3: Having enough time	Having time for restful breaks and to thoroughly complete any work.	I have enough time to think about things properly, and I don't feel time pressured.	13 (86.7%)	41 (26.1%)
Sub‐theme 1: Time for breaks	Any break during the day away from work or social pressure.	I might just need a bit of time away from everyone else in order to recharge so that I can put on a front for clients to act like a human.	11 (73.3%)	22 (14.0%)
Sub‐theme 2: A steady pace	Having enough time to do tasks properly without pressure from being rushed or over‐booked.	Not having other demands put on my time, having the time to do stuff, I took a lot longer to do stuff in practice than other people ‘cause I did them really thoroughly.	11 (73.3%)	20 (12.7%)
Theme 4: A sense of achievement	Feelings of success and self‐worth.	You get home and you feel like you've done it, you've done a good day's work.	11 (73.3%)	32 (20.4%)
Sub‐theme 1: Making a difference	Making a positive difference to the lives of clients and patients.	Save some animals or make some animals feel better, or even end some lives really nicely so that the owners are going to remember that it was a beautiful death.	6 (40.0%)	8 (5.10%)
Sub‐theme 2: Feeling accomplished	Successfully ending an exciting or challenging case and feeling accomplished.	A day where it's absolutely manic, but you've coped with it and you go home, you're tired, but you've got that positive ‘yes, we did it’ type attitude.	11 (73.3%)	27 (17.2%)

#### Theme 1: Positive interactions

This comprised two sub‐themes: connecting with colleagues and good client interactions. All the participants referred to the importance of positive communication and good relationships with both clients and colleagues on good workdays.

Being able to ‘connect’ and talk easily and freely to colleagues was important for reducing stress: ‘A good day's when the communication happens and is easy and I'm not feeling constantly like I'm having this speak another language to get them to understand what I'm asking or saying’ (participant 2). The participants preferred interactions talking about work or teaching rather than social chat.

Good client interactions intersect with connecting with colleagues. The interviewees described feeling ‘safe’ communicating with clients as they ‘put their vet‐head on’ (participant 1), playing a professional role with structured interactions, which is therefore less anxiety inducing: ‘I'm much more comfortable with communicating with clients, and I think it's because I'm in the role of the vet […] and automatically that puts me on much more confident footing’ (participant 8). Having a nurse in the consultation room to handle client emotions enabled some veterinary surgeons to feel more able to practice medicine: ‘I bring a nurse in to do the emotional bit and they can kind of do the client bit that I'm not very good at’ (participant 11).

#### Theme 2: Feeling in control

A feeling of control comprised two sub‐themes: the physical environment and a touchstone amidst the chaos. Despite being a broad theme, a clear preference for consistency, routine and a sense of control was evident.

The physical environment describes the preferences for a clean, organised practice environment that enabled participants to cope with other stressful elements of the day: ‘Controlling my environment to a degree makes a big difference … I'll just tidy things up, clean things, nothing drastic […] but all of those things mean then I don't have to think about them’ (participant 5).

A touchstone amidst the chaos describes how feeling in control allows participants to do their jobs more efficiently. Having autonomy over certain aspects of the day, such as their schedule, colleague interactions and having some predictable, routine cases, allowed management of other stress: ‘Vet medicine is never going to be predictable but keeping some things steady as a touchstone helps me at least deal with the chaos of the rest of the day’ (participant 2).

#### Theme 3: Having enough time

This was mentioned by almost all the participants and was emphasised by several as being the most important factor for a good day at work. This theme consists of two sub‐themes: time for breaks and a steady pace.

The time for breaks sub‐theme illustrated the importance of a restful break during the workday for reducing stress levels. Farm and equine veterinary surgeons reported using car journeys between clients as restorative: ‘I use that time when I'm driving between calls just to switch off’ (participant 3).

Working at a steady pace and having enough time to properly work on cases and consult with other veterinarians reduced stress. A principal element within this sub‐theme was being in control of the schedule, without too many interruptions or extra cases, allowing the necessary tasks to be done well: ‘Not having other demands put on my time, having the time to do stuff, I took a lot longer to do stuff than other people ‘cause I did them really thoroughly’ (participant 8).

#### Theme 4: A sense of achievement

Feelings of achievement and self‐worth played an important role in good days at work and comprised two sub‐themes: making a difference and feeling accomplished.

Making a difference included participants solving a good case or helping the lives of animals and clients. These feelings were associated not only with saving lives but also with giving animals a ‘dignified death’ through euthanasia: ‘That gives me quite a good feeling, a fulfilling feeling of satisfaction, like I made a difference’ (participant 12).

Feeling accomplished involves feelings of achievement after an exciting or challenging case. The act of acknowledging that they had done something well allowed the participants to enjoy their work more and reduced feelings of imposter syndrome: ‘We all like to feel that we're kind of having an impact, a positive impact in the role that we're doing’ (participant 1).

## DISCUSSION

This is the first study to elucidate factors deemed to play a role in a good or difficult workday for autistic veterinary surgeons. The wellbeing and psychosocial work environment of autistic veterinary surgeons in UK clinical practice is significantly lower than that of the wider UK veterinary population and general workforce norms.^7^ Moreover, psychosocial working conditions accounted for 44% of the unique variance in mental wellbeing of autistic veterinarians; therefore, these factors are important to consider when contemplating workplace modifications to improve the work experience of autistic veterinary employees.

Professional interactions were the only factor mentioned by all participants as pertinent to both good and difficult workdays, with negative colleague interactions twice as frequent as those involving clients. Likewise, communication challenges with peers were reported by medical doctors over three times more frequently than with communication challenges with patients.^6^ The stress of unstructured or social discussions with veterinary colleagues led participants to avoid such interactions, even if that meant missing out on rest time. Such self‐undermining behaviour, that is, decisions consciously taken to avoid difficult situations that result in poorer wellbeing, has been associated with increased impact of daily job demands and exhaustion.^17^

Similar to autistic doctors^6^ and artistic performers,^8^ participants frequently cited a lack of neurodiversity awareness among colleagues and microaggressions (indirect, subtle, or unintentional discrimination against members of a marginalised group) or discriminatory behaviour, highlighting the importance of improving education around neurodiversity and implementing a neuroinclusive culture in the workplace. Psychological safety is the most significant factor for success in high‐performing teams across many sectors,^18^ and neurodiversity awareness training for staff has been previously shown to improve job satisfaction in autistic employees.^19^, ^20^ Positive action in this regard is underway within the veterinary profession, as exemplified in the BVA's Great Workplace Scheme^21^ and Vetlife's 2024 Neurodiversity Awareness Campaign.^22^

Control of their schedule and the physical workspace were important factors influencing the experience of autistic veterinary surgeons. Uncertainty and unpredictability were frequently cited as important stressors, in agreement with a survey of 85 autistic veterinary surgeons, where role clarity and job control (decision latitude) were reported as organisational stressors significantly linked with mental wellbeing.^7^ Most participants experienced sensory overload at work, similar to autistic doctors^6^ and artistic performers.^8^ Schedule flexibility and changes to the physical environment (light or noise levels) have been previously reported to improve the work environment^19^ and have a strong relationship with job satisfaction for autistic employees.^23^ Environment audits can be useful to highlight sensory issues within a space, and design of inclusive workplaces is the focus of an ongoing campaign by Autistica.^24^

Factors identified as sources of stress in the general UK veterinary surgeon population include work‒life balance, interactions with animal owners, feeling unsafe when meeting clients alone, aspects of euthanasia, dealing with poor animal welfare, staff management responsibility, client complaints and on‐call work.^25^, ^26^, ^27^ Notably, interaction with colleagues, the unifying common factor for autistic veterinary surgeons, is absent from this list, which evidences the social communication differences experienced by autistic individuals. Organisation‐oriented interventions, including widening participation in practice decision making, provision of regular breaks and reducing working hours to improve wellbeing of veterinary surgeons, have previously been proposed.^28^ While these would benefit all veterinary surgeons, our study highlights the importance of a suitable space for autistic veterinary surgeons to allow adequate rest, which may be away from the social demands of being with other colleagues.

Autistic veterinary surgeons are entitled to reasonable accommodations under the Equality Act 2010. However, anecdotally, employers report a lack of knowledge and confidence in providing such support. Modification of environmental and external factors, including employer attitudes, the implementation of peer support networks and autism‐specific training, have been advocated to improve employment success and job satisfaction of autistic people.^6^, ^8^, ^29^, ^30^ In the current context of autistic veterinary surgeons, providing employers and managers with neurodiversity training and the knowledge required to make appropriate accommodations pertinent to the physical and psychological experience of the work environment may be meaningful for improving the wellbeing of autistic veterinarians. New resources have recently been made available within both veterinary and medical communities to facilitate this process.^22^ ^.^ ^31^

This study involved a small sample of self‐selected individuals with a strong female and companion animal sector bias. As such, the sector‐wide generalisability of these findings will require confirmation through a larger‐scale study. Qualitative research may not fully capture the context and subjective experiences of individuals and may be influenced by researcher bias. Although thematic saturation was reached during analysis, the limited number of interviews conducted may have led to missing perspectives of different groups, such as exotic animal veterinary surgeons and young, early‐career male veterinarians. Despite the predominant small animal sector representation, a large overlap in the stressors experienced by autistic veterinarians across small animal, farm and equine practice was apparent. Additionally, autistic veterinary surgeons not experiencing workplace challenges may not have been as motivated to participate.

In conclusion, good collegial relationships, control of sensory overload and maximising autonomy appear pivotal in enabling autistic veterinary surgeons in the workplace. Education to promote awareness and acceptance of neurodivergent differences in the workplace is key to improving the work experience of autistic veterinary surgeons.

## AUTHOR CONTRIBUTIONS

The initial study concept was Kirstie Pickles, with study design by Kirstie Pickles, Jonathan Houdmont and Bradley Hill. Data collection was undertaken by Femke Smits, with all the authors contributing to data analysis. All the authors contributed to the manuscript preparation and all the authors reviewed and approved the final manuscript.

## CONFLICT OF INTEREST STATEMENT

The authors declare no potential conflicts of interest with respect to the research, authorship and/or publication of this article.

## ETHICS STATEMENT

Ethical approval for the study was granted by the Research Ethics Committee of the School of Veterinary Medicine at the University of Nottingham (ref. 3369 210430).

## Supporting information



Supporting Information

## Data Availability

The data are available upon request due to privacy/ethical restrictions.
