# Supplementary material for: ‘Part of the team as opposed to watching from the outside’: Critical incident study of autistic veterinary surgeons’ workdays
Source: Vet Rec. 2024 Dec 2;196(4):e4957. doi: 10.1002/vetr.4957 (PMC11827692; doi:10.1002/vetr.4957)
Supplement: Supplementary file 1 — Supporting Information [file VETR-196-e4957-s001.docx]

Appendix A: Interview structure with prompts for critical incident study.

Thank you for participating in this study. We want to learn more about the characteristics which define a good and a difficult day at work for you. Take as much time as needed to respond thoroughly and accurately to the questions.

1. Demographic information:

a) How long have you been working in practice?

b) What kind of practice do you work in and what is your role?

c) How long has it been since you received a diagnosis?

2. How would you define a good day at work?

3. Can you recall a specific good day at work? Please can you describe this event including the preparation, during and post-event details?

a. Prompts

i. Can you describe the timeline of events that occurred starting from the preparation stage?

ii. Please can you describe the day from start to finish?

iii. What happened then?

iv. What environmental factors played a role? For example, lighting, noises, seating, weather.

v. What equipment factors played a role?

1. Were you comfortable with the equipment?

2. Was it working well?

vi. What human factors played a role?

1. Yourself

2. Owners

3. Colleagues

4. Managers

vii. What communication factors played a role?

viii. Any other factors? E.g., support from colleagues or managers

4. Can you recall a specific difficult day at work? Please can you describe this event including the preparation, during and post-event details?

a. Prompts

i. Can you describe the timeline of events that occurred starting from the preparation stage?

ii. Please can you describe the day from start to finish?

iii. What happened then?

iv. What environmental factors played a role? For example, lighting, noises, seating, weather.

v. What equipment factors played a role?

1. Were you comfortable with the equipment?

2. Was it working well?

vi. What human factors played a role?

1. Yourself

2. Owners

3. Colleagues

4. Managers

vii. What communication factors played a role?

viii. Any other factors? E.g., support from colleagues or managers

5. Is there anything else you would like to mention regarding these events?

6. Could you tell me about the support you have at work? Have you disclosed your diagnosis and if so, what support do you get?

Thank you for your time. We appreciate your participation in this study.
